# The Effect of Nonalcoholic Fatty Liver Disease on Extrahepatic Cancers: Evidence From Population‐Based Cohort and Mendelian Randomization

**DOI:** 10.1002/hsr2.70551

**Published:** 2025-03-10

**Authors:** Wei Wang, Pengfei Sun, Xintian Ren, Tingting Lv, Min Li

**Affiliations:** ^1^ Department of Clinical Laboratory, Beijing Friendship Hospital Capital Medical University Beijing China; ^2^ Department of Ultrasound, Beijing Friendship Hospital Capital Medical University Beijing China; ^3^ Clinical Epidemiology and EBM Unit, Beijing Friendship Hospital Capital Medical University Beijing China; ^4^ Liver Center, Beijing Friendship Hospital Capital Medical University Beijing China

**Keywords:** cancer, causality, epidemiology, Mendelian randomization, nonalcoholic fatty liver disease

## Abstract

**Background and Aims:**

Nonalcoholic fatty liver disease (NAFLD) is an escalating global health concern with significant implications for cancers. A better understanding of the causal relationship between NAFLD and extrahepatic cancers might help in clinical management of NAFLD and prevent its adverse outcomes.

**Methods:**

This study encompassed two complementary approaches. First, the cross‐sectional analysis was performed to examine the association between NAFLD and extrahepatic cancers, utilizing individual‐level data from the National Health and Nutrition Examination Survey (2017‐March 2020, 2021‐2023 cycles). Logistic regression model was utilized to evaluate the association. Subsequently, Mendelian randomization (MR) analysis was conducted to explore the causal association between NAFLD and extrahepatic cancers. Summary‐level data for genetically predicted NAFLD and extrahepatic cancers were derived from large‐scale genome‐wide association studies (GWAS), IEU Open GWAS project and the UK Biobank. The inverse variance weighting (IVW) method with a random‐effect model was utilized as the main analysis.

**Results:**

A total of 10,010 participants were included in the cross‐sectional analysis. No association was observed between NAFLD and extrahepatic cancers after adjusting for potential confounders, with odd ratios (ORs) ranging from 0.872 to 2.171. IVW MR analysis showed genetic liability to genetically predicted cALT and imaging‐and‐biopsy confirmed NAFLD were not causally associated with extrahepatic cancers, with ORs ranging from 0.957 to 1.118 (all *p* > 0.050). Moreover, genetically predicted cALT and imaging‐and‐biopsy confirmed NAFLD were causally associated with liver & bile duct cancer (OR = 1.001, 95% CI = 1.000–1.001, *p* = 0.011; OR = 1.001, 95% CI = 1.000–1.001, *p* < 0.001), reinforcing a well‐documented link between NAFLD and liver & bile duct cancer.

**Conclusion:**

Our findings demonstrated that NAFLD was not causally associated with common extrahepatic cancers. Further research is required to validate these results from a mechanistic perspective.

## 1 Introduction

Nonalcoholic fatty liver disease (NAFLD) impacts approximately 25% of people globally and is the primary contributor to chronic liver disease [1]. The prevalence of NAFLD is expected to increase steadily due to the obesity epidemic and the rising incidence of diabetes [2, 3]. NAFLD patients have higher risk of subsequent liver cancer and corresponding mortality than non‐NAFLD controls. Additional evidence indicates that NAFLD is linked to extrahepatic cancers, such as gastrointestinal, breast and gynecological cancers [4]. Nonetheless, there is a lack of robust evidence connecting NAFLD to extrahepatic cancers, with majority of current evidence stemming from observational studies that may be influenced by confounding variables.

Although NAFLD has been reported to have association with extrahepatic cancers, the causality of this association is uncertain. Mendelian randomization (MR) is an analytical approach used to evaluate the potential causality between exposure and outcome by utilizing genetic variants as instrumental variables [5]. Unlike observational studies, MR analysis could minimize confounding raised by some unmeasured factors when examining the association between NAFLD and extrahepatic cancers, owing to the random distribution of genetic variants at meiosis [6]. Besides, the one‐way relationship from DNA sequences to phenotypes minimizes the chance of reverse causality, since genetic variants are determined at birth and remain constant throughout life [7].

Up to date, limited MR study examining the association between NAFLD and extrahepatic cancers was performed due to a lack of comprehensive gene‐exposure datasets [8]. Recently, a comprehensive genome‐wide association study (GWAS) on chronically high serum alanine aminotransferase levels (cALT) was conducted with a sample size of 218,595, alongside studies on imaging‐confirmed and biopsy‐verified NAFLD [9]. The accessibility of this data enables additional MR analysis to be conducted. Herein, we analyzed data from 10,010 participants in the National Health and Nutrition Examination Survey (NHANES) to explore cross‐sectional association of NAFLD and extrahepatic cancers. Additionally, a two‐sample MR was performed to assess the causal association between NAFLD and extrahepatic cancers utilizing genetic variants that are strongly linked to NAFLD as instrumental variables.

## 2 Methods

### 2.1 Cross‐Sectional Analyses of NHANES Database

The NHANES is an ongoing national survey designed to assess the prevalence of major diseases, which employs a sophisticated, multistage probability sampling method that ensures the cohort's representativeness of noninstitutionalized US population [10]. We extracted deidentified data for participants aged ≥ 20 years older from the NHANES 2017‐March 2020, 2021–2023 cycles.

In this study, we selected controlled‐attenuation parameter (CAP) value to detect participants with hepatic steatosis, which have been developed and validated in the US population. An optimal CAP cutoff of ≥ 274 dB/m is indicative of hepatic steatosis. Disease status of cancer/malignancy was obtained through a positive answer to a relevant question, “Ever been told you have the corresponding disease?”

### 2.2 MR Analyses

The genetic variants closely linked to NAFLD were derived from a recent GWAS, in which NAFLD was characterized by elevated ALT levels above 40 U/L for males or 30 U/L for females at least twice and spaced a minimum of 6 months apart over 2 years, excluding other liver diseases [9]. This GWAS involved 90,408 cases of cALT and 128,187 controls sourced from the Million Veteran Program in the discovery cohort. After adjusting for factors like age, gender, age‐adjusted Alcohol Use Disorders Identification Test score, and the first ten principal components of genetic ancestry, 77 single nucleotide polymorphisms (SNPs) reached genome‐wide significance (*p* < 5 × 10^−8^). Of these, 17 SNPs were verified in two external validation cohorts: liver fat extracted from radiologic imaging (*n* = 44,289) and biopsy‐confirmed NAFLD (*n* = 64,182) [9].

The GWAS data for 18 types of cancers, including cancers of esophagus, stomach, colorectal, lung, pancreatic, thyroid, prostate, bladder, kidney, skin, malignant lymphoma, non‐Hodgin lymphoma, leukemia, breast, cervical, endometrial, ovarian, and liver & bile duct were obtained from published GWASs [11, 12, 13, 14, 15, 16], the Medical Research Council Integrative Epidemiology Unit Open GWAS project (https://gwas.mrcieu.ac.uk/), and UK Biobank study [17]. Table 1 provided details of the GWAS databases utilized in this study.

**Table 1:** Details of GWAS datasets of common type of cancers.

Cancer types	Cases	Controls	Sample size	Year	Population	Source/PMID
Esophagus cancer	2386	634,510	636,895	2021	European (Finland, UK) East Asian (Japan)	GCST90018841/34594039
Stomach cancer	8950	634,288	643,238	2021	European (Finland, UK) East Asian (Japan)	GCST90018849/34594039
Colorectal cancer	14,886	622,807	637,693	2021	European (Finland, UK) East Asian (Japan)	GCST90018808/34594039
Lung cancer	8235	663,294	671,529	2021	European (Finland, UK) East Asian (Japan)	GCST90018875/34594039
Pancreatic cancer	1695	634,250	635,945	2021	European (Finland, UK) East Asian (Japan)	GCST90018893/34594039
Thyroid cancer	1415	669,282	670,697	2021	European (Finland, UK) East Asian (Japan)	GCST90018929/34594039
Prostate cancer	79,148	61,106	140,254	2018	European (Canada, US, Australia, Belgium, France, Germany, Netherlands, Japan, Bulgaria, Poland, Denmark, Finland, Norway, Sweden, UK, Portugal, Spain)	GCST006085/29892016
Bladder cancer	1279	372,016	373,295	2021	European	ieu‐b‐4874
Kidney cancer	1338	410,350	411,688	2020	European (US, UK)	GCST90011818/32887889
Skin cancer	26,082	644,847	670,929	2021	European (Finland, UK) East Asian (Japan)	GCST90018921/34594039
Malignant Lymphoma	3881	664,599	668,480	2021	European (Finland, UK) East Asian (Japan)	GCST90018878/34594039
Non‐Hodgin Lymphoma	2400	410,350	412,750	2020	European (US, UK)	GCST90011819/32887889
Leukemia	1260	372,016	373,276	2021	European (UK Biobank)	ieu‐b‐4914
Breast cancer (Females)	122,977	105,974	228,951	2017	European	ieu‐a‐1126 (BCAC)/29059683
Cervical cancer (Females)	1889	461,044	462,933	2018	European	ukb‐b‐8777
Endometrial cancer	12,906	108,979	121,885	2018	European (US, Australia, Belgium, Germany, Norway, Sweden, UK)	GCST006464/30093612
Ovarian cancer (Females)	25,509	40,941	66,450	2017	European	ieu‐a‐1120 (OCAC)/28346442
Liver and bile duct cancer	350	372,016	372,366	2021	European (UK Biobank)	ieu‐b‐4915

Abbreviations: BCAC, breast cancer association consortium; GWAS, genome‐wide association study; OCAC, ovarian cancer association consortium.

**Figure 1:**
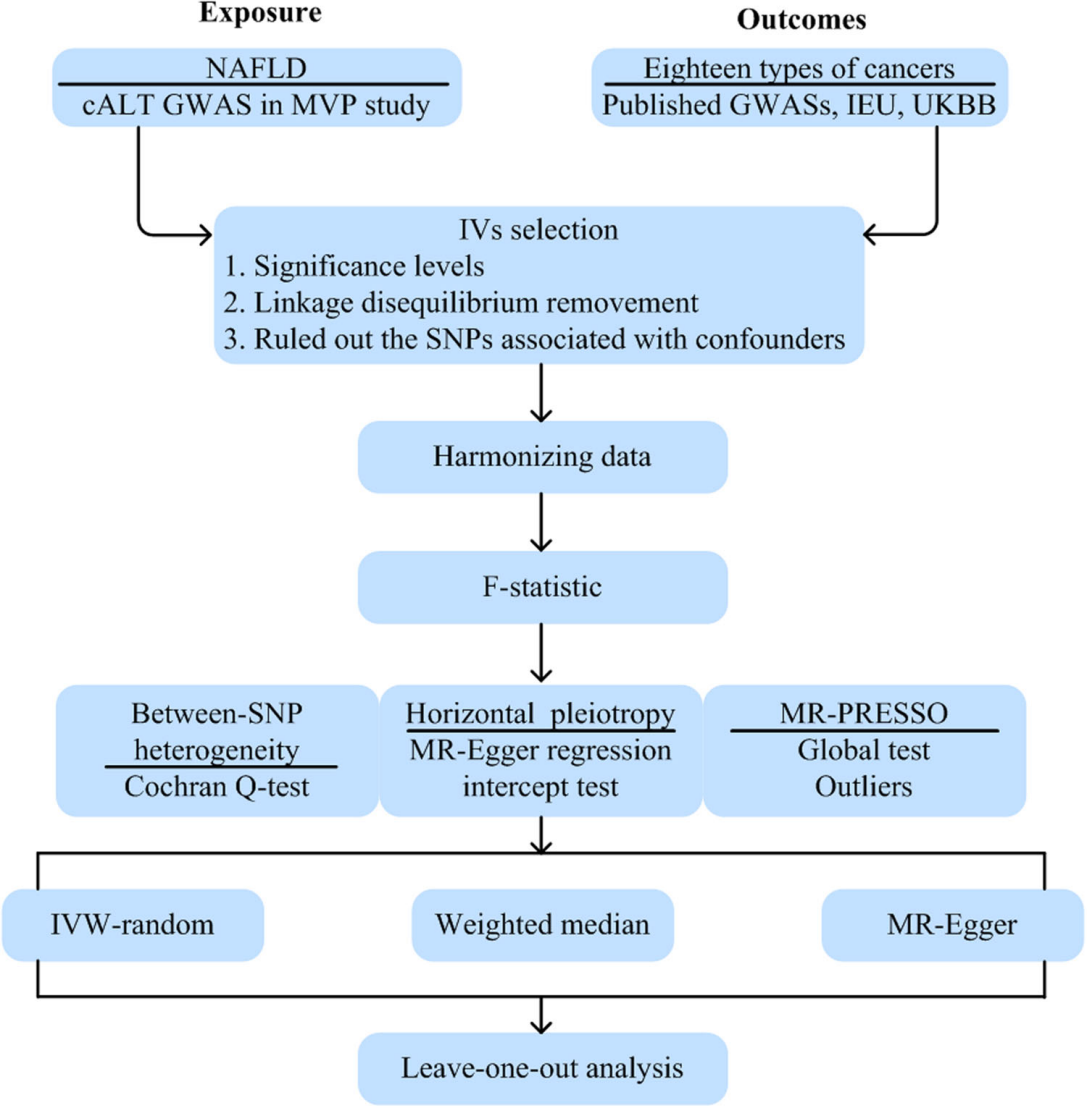
The flowchart of the current two‐sample Mendelian randomization analysis.

In the current MR analysis, two sets of instrumental variables served as genetic indicators for NAFLD: (1) all 77 SNPs associated with cALT, and (2) 17 SNPs validated in external histologic and/or radiologic NAFLD cohorts. The selected SNPs were clumped for independent inheritance with linkage disequilibrium (LD) *R* ^2^ < 0.001 as well as physical distance within 10000 kb. The remained SNPs were queried using the PhenoScanner database to exclude those linked to potential confounders like smoking, alcohol consumption frequency, and body mass index. Besides, SNPs were excluded if they were correlated with the outcomes of interest at *p* < 5 × 10^‐5^. Finally, we additionally harmonized the effect allele across the exposure and outcome datasets and excluded palindromic SNPs. Moreover, the effectiveness of the chosen genetic instruments was evaluated through F‐statistics, with F‐statistic exceeding 10 considered as a robust set of instrumental variables [18, 19] (Figure 1). Comprehensive details about the genetic variants utilized as instrumental variables in this analysis were shown in Tables S1 and S2.

### 2.3 Statistical Analyses

We examined the cross‐sectional association of NAFLD and cancers using logistic regression models. In multivariable analysis, a series of potential confounders such as age at recruitment, gender, race and ethnicity, education, family income, marital status, smoking, alcohol use, and medical history (including congestive heart failure, coronary heart disease, and stroke) were adjusted. Following the NHANES analytic guidelines, we incorporated the complex sampling design and sampling weights in our analyses to generate national estimates [20]. Sampling weight was reweighted in using combined NHANES cycles.

In MR analysis, the inverse variance weighting (IVW) method with a random‐effect model was utilized as the main analysis to estimate the causal association between NAFLD and cancers [21]. The weighted median and MR‐Egger methods were additional performed to examine the consistency of results [21]. The Cochran' *Q* statistic and *I* ^*2*^ was utilized to assess heterogeneity [22]. We used MR‐Egger intercept analysis to evaluate horizontal pleiotropy [23] and MR Pleiotropy Residual Sum and Outlier (MR‐PRESSO) to reveal the impact of outliers. Additionally, leave‐one‐out analysis was conducted to reveal the influence of any single SNP on results.

To account for the multiple testing, the Bonferroni correction threshold of *p* < 0.003 (0.050/18) was prespecified. *p* < 0.003 was considered significant, while *p* value ranging from 0.003 to 0.050 was viewed as indicative evidence. All analysis was conducted using R software (version 3.5.3).

## 3 Results

### 3.1 No Association Between NAFLD and Extrahepatic Cancers in Cross‐Sectional Analyses

Of 17,041 participants aged ≥ 20 years older, 3505 were excluded for not undergoing liver transient elastography and 8 were excluded for lacking cancer information. We also excluded 1477 participants with positive serum hepatitis B surface antigen/hepatitis C RNA, and another 2041 for excessive alcohol consumption. Consequently, the remaining 10,010 participants were included in this study.

Compared to non‐NAFLD participants, those with NAFLD were generally older, more likely to be male, had higher education levels, and were more often married and of Mexican American descent. Furthermore, participants with NAFLD exhibited higher rates of congestive heart failure, coronary heart disease, and stroke (Table 2).

**Table 2:** Baseline characteristics of the study participants by NAFLD.

Characteristics	NAFLD (*N* = 4362)	Non‐NAFLD (*N* = 5648)	*p* value
Age			< 0.001
20–29	300 (9.531) [8.274–10.789]	837 (20.351) [18.071–22.632]	
30–39	453 (13.147) [11.619–14.675]	791 (16.109) [14.859–17.359]	
40–49	650 (16.470) [14.754–18.187]	790 (15.291) [14.007–16.575]	
50–59	862 (22.109) [20.383–23.834]	831 (15.807) [14.205–17.408]	
≥ 60	2097 (38.743) [35.964–41.523]	2399 (32.442) [29.860–35.025]	
Gender			< 0.001
Male	2259 (54.757) [52.414–57.100]	2506 (44.353) [42.685–46.020]	
Female	2103 (45.243) [42.900–47.586]	3142 (55.648) [53.980–57.315]	
Race and ethnicity			< 0.001
Mexican American	504 (8.884) [6.430–11.338]	393 (5.697) [4.152–7.242]	
Other Hispanic	422 (7.695) [6.270–9.120]	534 (7.694) [6.173–9.216]	
Non‐Hispanic White	1986 (63.899) [59.974–67.823]	2434 (62.696) [58.715–66.676]	
Non‐Hispanic Black	814 (9.485) [7.378–11.592]	1377 (12.961) [10.579–15.343]	
Other	636 (10.037) [8.134–11.941]	910 (10.952) [9.009–12.895]	
Education level			0.023
High school or less	1763 (38.679) [35.381–41.976]	2126 (34.950) [32.027–37.872]	
Some college or higher	2592 (61.322) [58.025–64.619]	3517 (65.050) [62.128–67.973]	
Family income			0.406
Low	921 (15.943) [14.269–17.618]	1241 (16.166) [14.395–17.937]	
Medium	1484 (31.693) [28.765–34.621]	1812 (29.6062) [27.434–31.779]	
High	1365 (40.470) [37.077–43.863]	1811 (41.8400) [39.302–44.378]	
Marital status			< 0.001
Married	2662 (67.112) [63.874–70.351]	3114 (58.381) [56.031–60.731]	
Never married	1064 (19.202) [17.292–21.112]	1383 (20.154) [18.780–21.529]	
Living with partner	629 (13.619) [11.498–15.740]	1147 (21.426) [19.620–23.232]	
Other	7 (0.067) [0.003–0.131]	4 (0.040) [0.000–0.083]	
Smoke			0.001
Never	2600 (60.931) [58.092–63.770]	3504 (62.690) [60.409–64.972]	
Previous	1222 (27.927) [25.567–30.287]	1256 (23.471) [21.927–25.016]	
Current	539 (11.142) [9.461–12.824]	882 (13.838) [11.805–15.872]	
Alcohol drinker	3475 (81.027) [78.978–83.076]	4414 (81.610) [79.967–83.254]	0.173
Disease condition			
Congestive heart failure	193 (3.407) [2.620–4.195]	135 (1.527) [1.179–1.876]	< 0.001
Coronary heart disease	259 (5.773) [4.245–7.301]	220 (3.128) [2.385–3.872]	< 0.001
Stroke	223 (4.396) [3.684–5.107]	274 (3.245) [2.662–3.827]	0.042

*Note:* Data were presented as unweighted number and weighted percentage with 95% confidence interval.

Abbreviation: NAFLD, nonalcoholic fatty liver disease.

Participants with NAFLD exhibited a higher prevalence of thyroid and prostate cancers compared to non‐NAFLD participants (0.515% vs. 0.238%; 2.121% vs. 1.308%) (Table S3). For the association of NAFLD with thyroid and prostate cancers, the unadjusted odds ratios (ORs) were 2.169 (1.008–4.667) and 1.635 (1.091–2.451), respectively. However, these association became nonsignificant after adjusting for a series of covariates and Bonferroni correction, with ORs being 2.171 (1.035–4.557) and 1.242 (0.818–1.886), respectively (Table 3).

**Table 3:** Association between NAFLD and extraheptic cancers among the study participants.

Outcomes	Univariable analysis		Multivariable analysis	
	Crude OR (95%CI)	*p*	Adjusted OR^a^ (95%CI)	*p*
Esophagus cancer	1.162 (0.237–5.704)	0.850	1.062 (0.227–4.966)	0.939
Colorectal cancer	1.233 (0.634–2.399)	0.528	1.061 (0.605–1.858)	0.837
Lung cancer	1.213 (0.558–2.633)	0.618	0.982 (0.439–2.197)	0.965
Pancreatic cancer	2.360 (0.200–27.851)	0.486	1.654 (0.184–14.852)	0.653
Thyroid cancer	2.169 (1.008–4.667)	0.048	2.171 (1.035–4.557)	0.040
Prostate cancer	1.635 (1.091–2.451)	0.019	1.242 (0.818–1.886)	0.308
Bladder cancer	1.735 (0.819–3.674)	0.146	1.251 (0.591–2.648)	0.559
Kidney cancer	1.346 (0.589–3.073)	0.472	1.086 (0.479–2.459)	0.844
Skin cancer	1.088 (0.836–1.416)	0.521	0.872 (0.670–1.135)	0.309
Hodgin Lymphoma	1.957 (0.808–4.739)	0.133	1.711 (0.717–4.081)	0.226
Leukemia	0.805 (0.303–2.141)	0.657	0.774 (0.292–2.049)	0.606
Breast cancer	1.241 (0.914–1.684)	0.161	1.276 (0.914–1.783)	0.153
Cervical cancer	0.677 (0.380–1.204)	0.179	0.695 (0.384–1.257)	0.228
Ovarian cancer	1.470 (0.461–4.686)	0.506	1.507 (0.476–4.769)	0.485
Liver cancer	2.993 (0.387–23.135)	0.279	2.092 (0.295–14.819)	0.460

Abbreviations: CI, confidence interval; NAFLD, nonalcoholic fatty liver disease; OR, odd ratio.

^a^ Adjusted for socio‐demographic variables (age, gender, race and ethnicity, educational, family income, marital status), medical history (congestive heart failure, coronary heart disease, stroke), smoking, and alcohol drinking status.

### 3.2 No Causal Impact of NAFLD on Extrahepatic Cancers in MR Analyses

**Figure 2:**
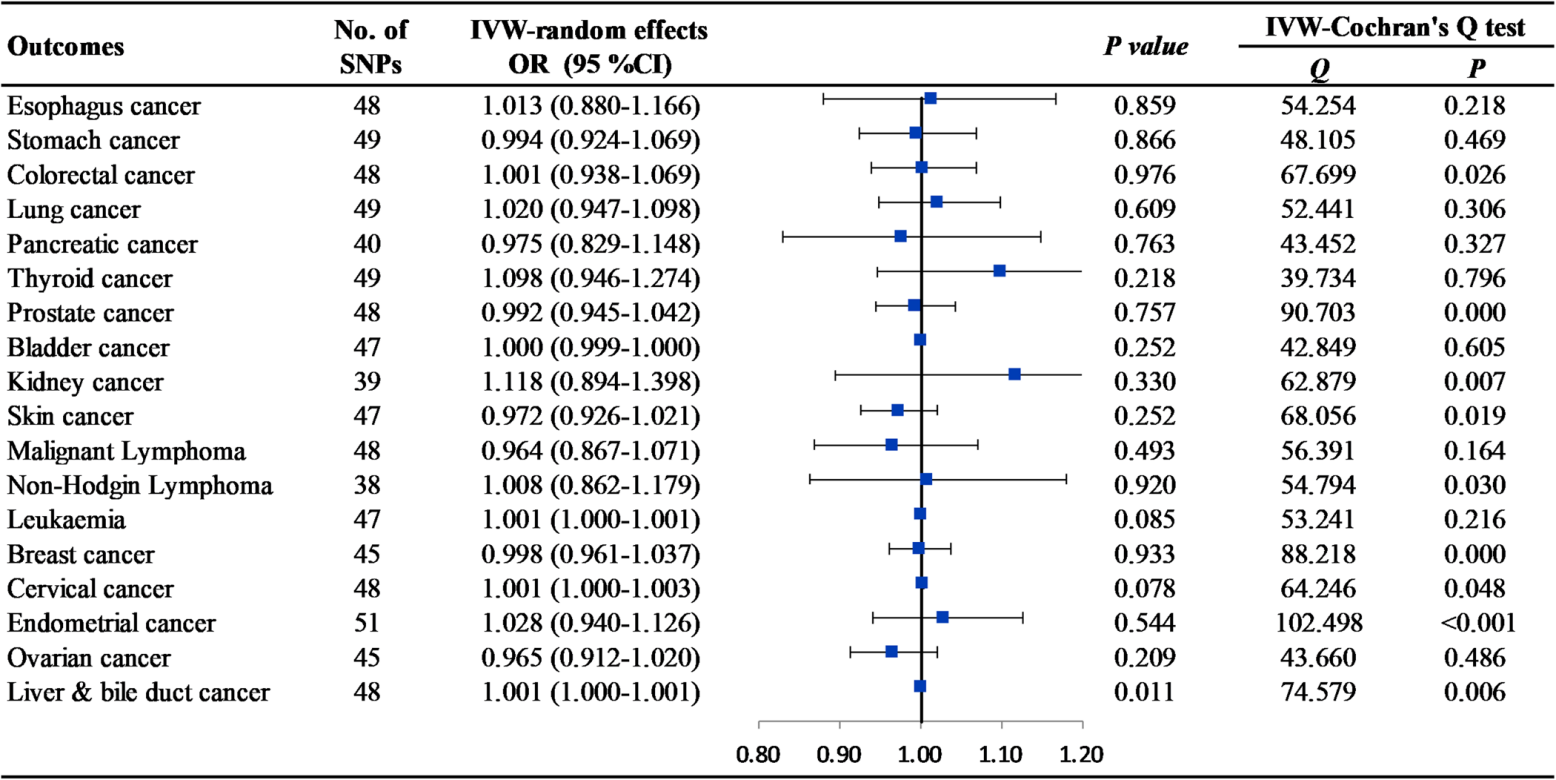
The causal association between genetically predicted cALT with extrahepatic cancers using the inverse variance weighted (IVW) method.

**Figure 3:**
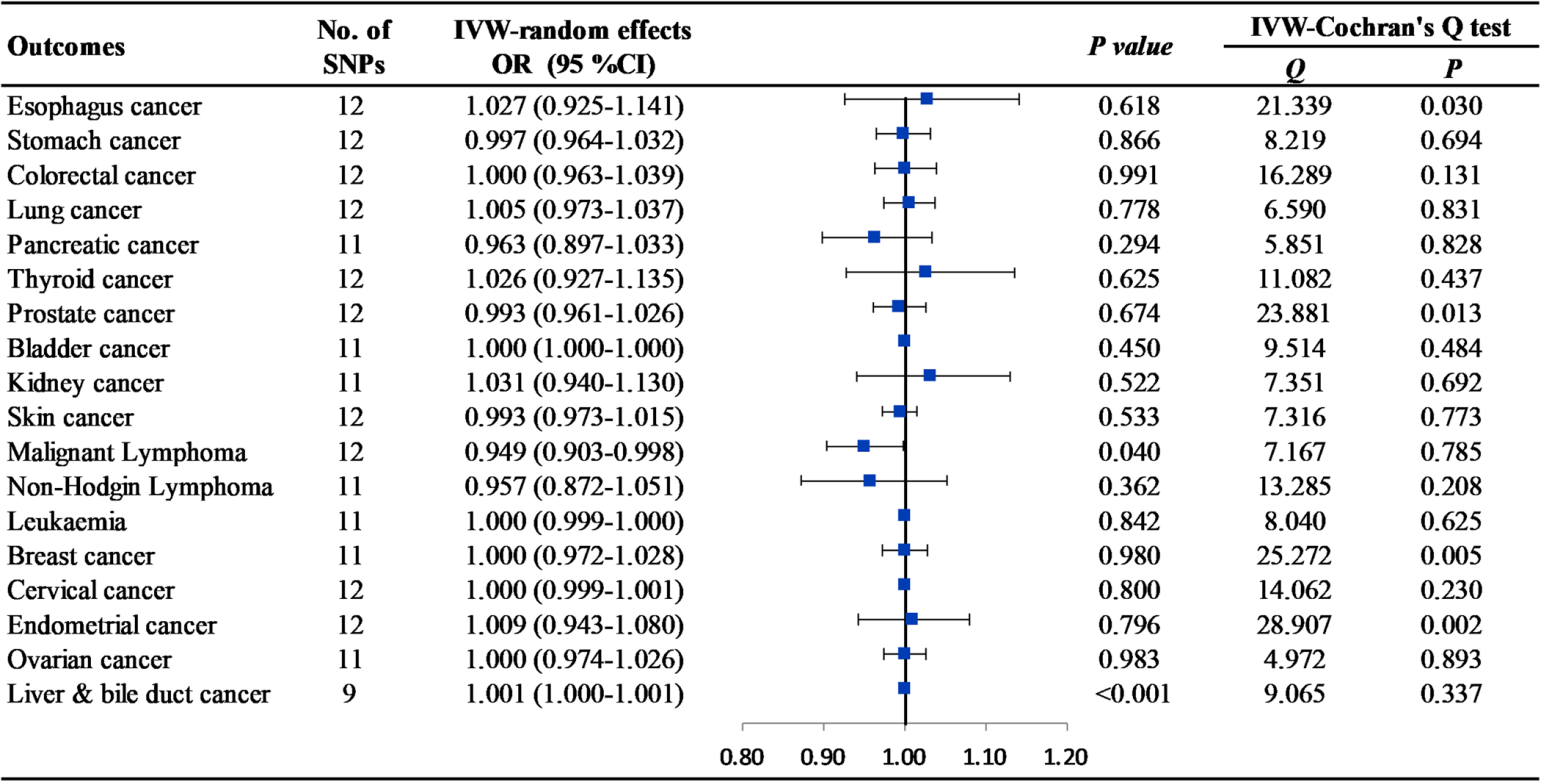
The causal association between genetically predicted imaging‐and‐biopsy confirmed NAFLD with extrahepatic cancers using the inverse variance weighted (IVW) method.

The relevant SNPs utilized as genetic instruments exhibited a median F‐statistic of 51 (minimum 27), indicating strong association between the selected genetic instruments and NAFLD. In the absence of horizontal pleiotropy, the primary MR method was the IVW approach using a random‐effects model. Our findings suggested that genetically predicted cALT was not causally associated with any type of extrahepatic cancers, with ORs ranging from 0.964 to 1.118 (all *p* > 0.050) (Figures 2 and S1). Similar results were obtained when only SNPs linked to imaging‐and‐biopsy confirmed NAFLD, which were significant and consistent in direction across both methods, were analyzed (Figures 3 and S2) (Tables S4 and S5).

Additionally, we found that genetic liabilities to genetically predicted cALT was suggestive causally associated with increased risk of liver and bile duct cancer (OR = 1.001, 95% confidence interval [CI] = 1.000–1.001, *p* = 0.011) (Figures 2 and S1). Moreover, the imaging‐and‐biopsy confirmed NAFLD was causally linked to liver and bile duct cancer at significant level (OR = 1.001, 95% CI = 1.000–1.001, *p* < 0.001), supporting a well‐established association between NAFLD and liver and bile duct cancer (Figures 3 and S2).

### 3.3 Robustness of MR Results

No horizontal pleiotropy was observed for most cancers, as the *p* values for MR‐Egger regression intercept were all greater than 0.050. Although the association between NAFLD and esophageal cancer showed signs of horizontal pleiotropy, the MR‐Egger analysis indicated no causal link between genetically predicted cALT and both imaging‐ and biopsy‐confirmed NAFLD with esophageal cancer. Furthermore, we found evidence of heterogeneity between genetically predicted cALT and cancers of colorectal, prostate, kidney, skin, non‐Hodgin Lymphoma, breast, cervical, endometrial, and liver & bile duct, and between imaging‐and‐biopsy confirmed NAFLD and cancers of esophagus, prostate, breast, and endometrial (Tables S6 and S7). However, consistent results were observed utilizing IVW, MR‐Egger, and weighted median method, indicating the robustness of our results (Tables S4 and S5). Additionally, Funnel plots and leave‐one‐out analysis were performed and showed in Figures S3–S6.

## 4 Discussion

This study combined epidemiological and MR analyses to evaluate the association between NAFLD and extrahepatic cancers. No causal association between NAFLD and extrahepatic cancers were observed in analyzing individual data and MR analysis. However, the MR analysis revealed causal association between genetic susceptibility to NAFLD and liver and bile duct cancer, reinforcing substantial evidence from observational studies that NAFLD could increase the risk of liver & bile duct cancer.

Liver cancer significantly contributes to cancer‐related deaths and illnesses worldwide. At present, NAFLD is responsible for 1%–38% of liver cancer across various regions [24]. Moreover, the rise in liver cancer linked to NAFLD is expected to persist as the rates of obesity and diabetes continue to grow. As supported by plenty of studies, NAFLD is widely recognized as a significant risk factor for liver cancer [25, 26, 27]. This MR analysis has validated this association, demonstrating that NAFLD is causally linked to liver & bile duct cancer. Although higher prevalence of liver cancer was observed in NAFLD participants, the association was not significant in analyzing individual data. The possible explanation might be that participants with higher risk of liver cancer (those with excessive alcohol consumption, hepatitis B and hepatitis C) were excluded in our study, resulting in fewer cases of liver cancer observed.

Currently, the association between NAFLD and extrahepatic cancers has not been thoroughly established. Some observational studies have suggested that NAFLD contributed to an increased risk of certain extrahepatic cancers, such as gastrointestinal, breast, and kidney cancers [28, 29, 30]. Zhou et al. conducted a meta‐analysis of observational studies and found that metabolic dysfunction‐associated steatotic liver disease was associated with an elevated risk of gastric, colorectal, pancreatic, biliary duct, thyroid, urinary system, breast, skin, and female genital cancers [31]. However, this meta‐analysis reported moderate to high heterogeneity due to differences in study design, patient characteristics, and outcomes. Although all the studies accounted for confounding factors, the specific adjustments made varied, and some studies did not fully address common risk factors. In contrast, our analysis using NHANES data demonstrated that NAFLD was not associated with common extrahepatic cancers. One possible explanation for this discrepancy is that our study was based on a US population, whereas the majority of studies included in the meta‐analysis were from Asian countries. Given the differences in body fat distribution, genetic and cultural backgrounds, and lifestyle habits between Asian and non‐Asian populations, these factors may influence the association between NAFLD and extrahepatic cancers. Moreover, similar to the meta‐analysis, our study cannot entirely rule out the possibility of residual confounding due to unmeasured factors.

Given the constraints of observational studies in determining causal association, we conducted MR analyses to explore the causal link between NAFLD and extrahepatic cancers. However, our MR analysis did not identify any causal association between genetic susceptibility to NAFLD and extrahepatic cancers. It is possible that the association observed in observational studies may largely stem from shared risk factors or confounding variables. However, inconsistent with our results, a previous MR study aiming to reveal the causal association between NAFLD and extrahepatic cancers reported that NAFLD was causally associated with breast, cervical, laryngeal, leukemia, lung, and prostate cancers [8]. The potential explanation for the inconsistence is that our study employed gene‐exposure data from two distinct NAFLD‐related traits: genetically predicted cALT and imaging‐ and biopsy‐confirmed NAFLD, rather than relying on ICD codes. Additionally, we applied strict SNP selection criteria (*p* < 5 × 10^−8^) to minimize weak instrumental bias and included a larger number of SNPs as genetic instruments. Thus, the present study employed more rigorous analytical methods to investigate the causal association between genetically predicted NAFLD and extrahepatic cancers.

The potential mechanism underlying the increased risk of extrahepatic cancers associated with NAFLD has not been elucidated. The proposed potential pathophysiological mechanisms linking two diseases include an inflammatory state, insulin resistance, gut microbiota dysfunction, and adipose tissue dysfunction [3, 32, 33]. While both conditions share common metabolic risk factors, such as obesity and diabetes, it is uncertain whether the observed risk is solely due to these shared factors or if NAFLD itself directly contributes to the development of extrahepatic cancers, independent of these factors. An alternative hypothesis suggests that the association between NAFLD and extrahepatic cancers may primarily be due to the links with metabolic risk factors, which may account for the majority of the impact of NAFLD on cancer risk [34]. The strong association between NAFLD, obesity, diabetes, and insulin resistance complicates the task of determining the exact causal relationship between NAFLD and the increased risk of extrahepatic cancers.

The current study has some notable advantages. The key merit is the joint use of epidemiology data and MR approach, which strengthen causal inference by minimizing certain confounders typically raised in observational studies. Additionally, multiple SNPs were selected as genetic instruments in MR analysis by using large‐scale summary data, which strengthened our ability to demonstrate causal effect of NAFLD on extrahepatic cancers. We also excluded SNPs linked to potential confounders, including smoking, alcohol intake frequency, and body mass index. Besides, the consistent directional outcomes of all three MR methods demonstrated the reliability of our results. Nonetheless, our study has several limitations, including small sample sizes for certain cancer types, such as liver, leukemia, and bladder cancer. Additionally, because we used summary‐level data in the MR analysis, subgroup analyses based on factors like age, sex, or ethnicity could not be performed.

## Author Contributions

**Wei Wang:** writing – original draft, formal analysis. **Pengfei Sun:** writing – original draft. **Xintian Ren:** formal analysis. **Tingting Lv:** writing – review and editing. **Min Li:** conceptualization, writing – review and editing, funding acquisition, supervision.

## Ethics Statement

The NHANES study received approval from the National Center for Health Statistics Research Ethics Review Board. All participants provided written informed consent. The MR analysis utilized publicly accessible summary‐level data sourced from various large‐scale cohorts. The informed consent procedures are detailed in these cohorts. No further ethical approval was required for this study.

## Conflicts of Interest

The authors declare no conflicts of interest.

## Transparency Statement

The lead author Wei Wang affirms that this manuscript is an honest, accurate, and transparent account of the study being reported; that no important aspects of the study have been omitted; and that any discrepancies from the study as planned (and, if relevant, registered) have been explained.

## Supporting information

Supporting information.

Supporting information.

## Data Availability

The datasets in NHANES study is publicly accessibility: https://www.cdc.gov/nchs/nhanes/index.htm. The GWAS data are available through the following resources: (i) NHGRI‐EBI GWAS Catalog (http://www.ebi.ac.uk/gwas/), (ii) IEU open GWAS project (https://gwas.mrcieu.ac.uk/), and (iii) UK Biobank (http://www.nealelab.is/uk-biobank). The MR‐base IDs were listed in the Table 1.
