# Supplementary material for: The Effect of Nonalcoholic Fatty Liver Disease on Extrahepatic Cancers: Evidence From Population‐Based Cohort and Mendelian Randomization
Source: Health Sci Rep. 2025 Mar 10;8(3):e70551. doi: 10.1002/hsr2.70551 (PMC11892145; doi:10.1002/hsr2.70551)
Supplement: Supplementary file 1 — Supporting information. [file HSR2-8-e70551-s002.docx]

**The effect of nonalcoholic fatty liver disease on extrahepatic cancers: evidence from population-based cohort and Mendelian randomization**

Wei Wang ^1^, Pengfei Sun ^2^, Xintian Ren ^3^, Tingting Lv ^4^, Min Li ^3*^

**Affiliations:**

^1^ Department of Clinical Laboratory, Beijing Friendship Hospital, Capital Medical University, Beijing, China;

^2^ Department of Ultrasound, Beijing Friendship Hospital, Capital Medical University, Beijing, China;

^3^ Clinical Epidemiology and EBM Unit, Beijing Friendship Hospital, Capital Medical University, Beijing, China;

^4^ Liver Center, Beijing Friendship Hospital, Capital Medical University, Beijing, China;

Wei Wang and Pengfei Sun contributed equally.

***Correspondence author:** Min Li, Ph.D. Clinical Epidemiology and EBM Unit, Beijing Friendship Hospital, Capital Medical University, No. 95 Yong-an Road, Xi-Cheng District, Beijing 100050, China. E-mail: [mli@ccmu.edu.cn](mailto:mli@ccmu.edu.cn). Tel: 010-63139364.

**Contents**

**Supplemental Table 1.** Instrumental variables used in the MR analysis of genetically predicted cALT with extrahepatic cancers

**Supplemental Table 2.** Instrumental variables used in the MR analysis of genetically predicted imaging-based and biopsy-confirmed NAFLD with extrahepatic cancers

**Supplemental Table 3.** Prevalence of extraheptic cancers among the study participants, stratified by NAFLD

**Supplemental Table 4**. Causal effect of genetically predicted cALT on extraheptic cancers

**Supplemental Table 5**. Causal effect of genetically predicted imaging-based and biopsy-confirmed NAFLD on extraheptic cancers

**Supplemental Table 6**. Sensitivity analyses of MR analysis of genetically predicted cALT on extrahepatic cancers

**Supplemental Table 7**. Sensitivity analyses of MR analysis of genetically predicted imaging-based and biopsy-confirmed NAFLD on extrahepatic cancers

**Supplemental Figure 1**. Scatter plots of the causal effect of genetically predicted cALT on extrahepatic cancers

**Supplemental Figure 2**. Scatter plots of the causal effect of genetically predicted imaging-based and biopsy-confirmed NAFLD on extrahepatic cancers

**Supplemental Figure 3**: Funnel plots of the causal effect of genetically predicted cALT on extrahepatic cancers.

**Supplemental Figure 4**: Funnel plots of the causal effect of genetically predicted imaging-based and biopsy-confirmed NAFLD on extrahepatic cancers

**Supplemental Figure 5**: Leave-one-out analyses for genetically predicted cALT on extrahepatic cancers

**Supplemental Figure 6**: Leave-one-out analyses for genetically predicted imaging-based and biopsy-confirmed NAFLD on extrahepatic cancers

Supplemental Table 1. Instrumental variables used in the MR analysis of genetically predicted cALT with extrahepatic cancers

| SNP | Chromosome | Position | Beta of exposure | Standard error of exposure | P value of exposure | Effect allele of exposure | Other allele of exposure | Effect allele frequency of exposure |
| --- | --- | --- | --- | --- | --- | --- | --- | --- |
| rs2642438 | 1 | 220970028 | -0.075 | 0.007 | 6.65E-24 | A | G | 0.274 |
| rs6734238 | 2 | 113841030 | -0.057 | 0.006 | 4.94E-19 | G | A | 0.407 |
| rs13389219 | 2 | 165528876 | -0.053 | 0.007 | 8.20E-16 | T | C | 0.432 |
| rs17036160 | 3 | 12329783 | -0.068 | 0.011 | 3.39E-10 | T | C | 0.115 |
| rs10433937 | 4 | 88230100 | -0.081 | 0.008 | 5.28E-26 | G | T | 0.255 |
| rs17598226 | 4 | 100496891 | -0.042 | 0.007 | 5.61E-09 | G | C | 0.261 |
| rs4841132 | 8 | 9183596 | 0.123 | 0.011 | 6.62E-32 | A | G | 0.106 |
| rs2980888 | 8 | 126507308 | 0.130 | 0.007 | 4.21E-72 | T | C | 0.286 |
| rs10883451 | 10 | 101924418 | -0.152 | 0.007 | 2.65E-112 | C | T | 0.437 |
| rs4918722 | 10 | 113947040 | 0.077 | 0.008 | 1.75E-23 | C | T | 0.277 |
| rs28929474 | 14 | 94844947 | 0.492 | 0.027 | 9.01E-73 | T | C | 0.017 |
| rs56094641 | 16 | 53806453 | 0.042 | 0.007 | 1.36E-09 | G | A | 0.377 |
| rs1801689 | 17 | 64210580 | 0.170 | 0.019 | 1.46E-18 | C | A | 0.031 |
| rs11668950 | 19 | 18282940 | 0.046 | 0.007 | 2.22E-10 | A | G | 0.258 |
| rs58542926 | 19 | 19379549 | 0.209 | 0.013 | 6.54E-62 | T | C | 0.070 |
| rs5117 | 19 | 45418790 | -0.072 | 0.008 | 2.21E-20 | C | T | 0.231 |
| rs738408 | 22 | 44324730 | 0.267 | 0.008 | 3.99E-273 | T | C | 0.239 |
| rs36086195 | 1 | 16510894 | -0.043 | 0.007 | 4.50E-10 | C | T | 0.475 |
| rs79598313 | 1 | 27284913 | 0.172 | 0.024 | 4.91E-13 | T | C | 0.021 |
| rs74816838 | 1 | 161643560 | 0.078 | 0.012 | 1.51E-10 | T | C | 0.106 |
| rs1337101 | 1 | 219726100 | -0.052 | 0.007 | 1.98E-12 | T | G | 0.297 |
| rs848559 | 2 | 36694497 | -0.055 | 0.010 | 1.58E-08 | T | A | 0.141 |
| rs73024760 | 2 | 169885122 | 0.104 | 0.016 | 7.59E-11 | T | C | 0.045 |
| rs2943652 | 2 | 227108446 | -0.062 | 0.007 | 1.04E-19 | C | T | 0.353 |
| rs7653249 | 3 | 136005792 | -0.076 | 0.008 | 3.51E-22 | C | G | 0.229 |
| rs4683438 | 3 | 142652559 | -0.047 | 0.007 | 4.73E-12 | T | G | 0.338 |
| rs686250 | 6 | 32585055 | -0.039 | 0.007 | 3.06E-09 | A | G | 0.415 |
| rs4711750 | 6 | 43757082 | 0.037 | 0.007 | 4.79E-08 | A | T | 0.465 |
| rs4734654 | 8 | 103669991 | -0.044 | 0.007 | 1.55E-10 | G | A | 0.342 |
| rs2737217 | 8 | 116630311 | 0.044 | 0.007 | 3.92E-10 | A | G | 0.392 |
| rs7041363 | 9 | 117146043 | -0.130 | 0.007 | 1.02E-81 | G | C | 0.459 |
| rs687621 | 9 | 136137065 | 0.043 | 0.007 | 7.91E-11 | G | A | 0.354 |
| rs11601507 | 11 | 5701074 | 0.099 | 0.013 | 1.53E-14 | A | C | 0.072 |
| rs146774114 | 12 | 49743142 | -0.157 | 0.028 | 2.50E-08 | A | G | 0.015 |
| rs9668670 | 12 | 53278512 | -0.054 | 0.007 | 2.89E-15 | T | A | 0.346 |
| rs34123446 | 12 | 122511238 | -0.043 | 0.007 | 2.82E-11 | G | A | 0.444 |
| rs11621792 | 14 | 24871926 | 0.042 | 0.007 | 5.48E-10 | T | C | 0.415 |
| rs3935942 | 15 | 73971361 | 0.055 | 0.007 | 2.72E-15 | A | C | 0.384 |
| rs4782568 | 16 | 83980529 | -0.064 | 0.007 | 8.82E-21 | G | C | 0.420 |
| rs8082024 | 17 | 47945460 | 0.042 | 0.007 | 2.32E-08 | C | T | 0.322 |
| rs2207132 | 20 | 39142516 | 0.178 | 0.028 | 1.58E-10 | A | G | 0.028 |
| rs132665 | 22 | 36564170 | -0.066 | 0.010 | 1.18E-11 | G | A | 0.145 |
| rs6541349 | 1 | 93787867 | 0.049 | 0.008 | 1.76E-09 | C | T | 0.268 |
| rs6543007 | 2 | 101663584 | 0.036 | 0.007 | 5.00E-08 | T | C | 0.431 |
| rs11683409 | 2 | 112770134 | 0.044 | 0.007 | 9.19E-12 | G | C | 0.410 |
| rs10201587 | 2 | 202202791 | -0.044 | 0.006 | 4.18E-12 | G | A | 0.477 |
| rs11683367 | 2 | 233510011 | -0.057 | 0.007 | 6.38E-18 | C | T | 0.402 |
| rs934295 | 3 | 149122431 | 0.058 | 0.007 | 2.93E-16 | A | T | 0.360 |
| rs61791108 | 3 | 170732742 | 0.111 | 0.019 | 2.73E-09 | A | G | 0.030 |
| rs574044675 | 3 | 172274232 | -0.235 | 0.026 | 1.96E-19 | C | A | 0.023 |
| rs12500824 | 4 | 77416627 | 0.047 | 0.007 | 7.92E-13 | A | G | 0.391 |
| rs138033684 | 6 | 71895252 | 0.677 | 0.112 | 1.42E-09 | G | T | 0.006 |
| rs799165 | 7 | 73052057 | 0.060 | 0.010 | 1.32E-09 | A | T | 0.125 |
| rs115038698 | 7 | 87024718 | 0.642 | 0.068 | 3.51E-21 | T | C | 0.012 |
| rs4484649 | 8 | 10571491 | 0.045 | 0.007 | 1.38E-11 | C | A | 0.419 |
| rs141505249 | 8 | 145732114 | -2.020 | 0.123 | 7.15E-61 | C | G | 0.003 |
| rs35199395 | 10 | 70983936 | -0.039 | 0.007 | 2.79E-08 | G | C | 0.411 |
| rs148337160 | 10 | 104166504 | 0.088 | 0.013 | 2.89E-11 | C | T | 0.063 |
| rs174535 | 11 | 61551356 | -0.064 | 0.007 | 1.59E-20 | C | T | 0.336 |
| rs56175344 | 11 | 93864393 | -0.128 | 0.010 | 4.60E-40 | G | C | 0.124 |
| rs1626329 | 12 | 121622023 | 0.050 | 0.007 | 2.61E-14 | T | C | 0.388 |
| rs2296285 | 13 | 29009673 | 0.039 | 0.007 | 1.76E-08 | A | T | 0.335 |
| rs340009 | 15 | 60899639 | 0.043 | 0.007 | 3.65E-11 | A | C | 0.399 |
| rs7168849 | 15 | 90346227 | -0.064 | 0.009 | 1.67E-11 | G | A | 0.235 |
| rs12149380 | 16 | 72043546 | 0.048 | 0.008 | 2.25E-09 | G | C | 0.228 |
| rs7599 | 19 | 36038390 | 0.046 | 0.007 | 1.05E-12 | A | G | 0.388 |
| rs6059896 | 20 | 33111783 | 0.047 | 0.006 | 1.80E-13 | C | T | 0.485 |
| rs1547014 | 22 | 29100711 | -0.064 | 0.007 | 1.57E-21 | T | C | 0.342 |
| rs1047891 | 2 | 211540507 | 0.037 | 0.007 | 2.76E-08 | A | C | 0.325 |
| rs3852142 | 5 | 55796968 | -0.072 | 0.013 | 1.90E-08 | T | A | 0.077 |
| rs60315134 | 8 | 8670599 | 0.036 | 0.007 | 2.88E-08 | G | A | 0.457 |
| rs1658943 | 9 | 6676953 | -0.049 | 0.009 | 3.54E-08 | T | C | 0.158 |
| rs10774625 | 12 | 111910219 | 0.039 | 0.007 | 9.75E-09 | A | G | 0.465 |
| rs2727324 | 17 | 61922102 | 0.038 | 0.007 | 1.24E-08 | C | G | 0.368 |
| rs3810367 | 19 | 4342847 | 0.037 | 0.007 | 3.77E-08 | G | T | 0.374 |
| rs8108722 | 19 | 10347084 | 0.044 | 0.008 | 2.25E-08 | T | C | 0.220 |
| rs4805033 | 19 | 33839554 | -0.037 | 0.007 | 3.06E-08 | G | A | 0.439 |

Abbreviations: MR, mendelian randomization; cALT, chronically elevated serum alanine aminotransferase; SNPs, single nucleotide polymorphisms.

Supplemental Table 2. Instrumental variables used in the MR analysis of genetically predicted imaging-based and biopsy-confirmed NAFLD with extrahepatic cancers*

| SNP | Chromosome | Position | Beta of exposure | Standard error of exposure | P value of exposure | Effect allele of exposure | Other allele of exposure | Effect allele frequency of exposure |
| --- | --- | --- | --- | --- | --- | --- | --- | --- |
| rs11668950 | 19 | 18282940 | 0.106 | 0.026 | 4.07E-05 | A | G | 0.258 |
| rs1337101 | 1 | 219726100 | -0.055 | 0.027 | 4.40E-02 | T | G | 0.297 |
| rs13389219 | 2 | 165528876 | -0.095 | 0.023 | 3.86E-05 | T | C | 0.432 |
| rs17036160 | 3 | 12329783 | -0.082 | 0.036 | 2.50E-02 | T | C | 0.115 |
| rs17598226 | 4 | 100496891 | -0.112 | 0.026 | 1.45E-05 | G | C | 0.261 |
| rs1801689 | 17 | 64210580 | 0.260 | 0.065 | 5.65E-05 | C | A | 0.031 |
| rs2207132 | 20 | 39142516 | 0.297 | 0.088 | 6.90E-04 | A | G | 0.028 |
| rs2642438 | 1 | 220970028 | -0.180 | 0.027 | 1.50E-11 | A | G | 0.274 |
| rs2943652 | 2 | 227108446 | -0.062 | 0.023 | 6.00E-03 | C | T | 0.353 |
| rs2980888 | 8 | 126507308 | 0.178 | 0.025 | 2.42E-12 | T | C | 0.286 |
| rs3935942 | 15 | 73971361 | 0.054 | 0.022 | 1.50E-02 | A | C | 0.384 |
| rs4918722 | 10 | 113947040 | 0.127 | 0.025 | 4.18E-07 | C | T | 0.277 |
| rs5117 | 19 | 45418790 | -0.155 | 0.029 | 1.05E-07 | C | T | 0.231 |
| rs56094641 | 16 | 53806453 | 0.089 | 0.024 | 2.39E-04 | G | A | 0.377 |
| rs58542926 | 19 | 19379549 | 0.532 | 0.042 | 8.23E-37 | T | C | 0.070 |
| rs7041363 | 9 | 117146043 | -0.062 | 0.023 | 7.00E-03 | G | C | 0.459 |
| rs738408 | 22 | 44324730 | 0.686 | 0.025 | 9.96E-161 | T | C | 0.239 |

*including cALT SNPs that were directionally and nominally significant in both the imaging and biopsy cohort.

Abbreviations: MR, mendelian randomization; NAFLD, nonalcoholic fatty liver disease; SNPs, single nucleotide polymorphisms.

Supplemental Table 3. Prevalence of extraheptic cancers among the study participants, stratified by NAFLD

| Characteristics | NAFLD | Non-NAFLD | P value |
| --- | --- | --- | --- |
|  | (N=4362) | (N=5648) |  |
| Esophagus cancer | 3 (0.046) [0.000-0.102] | 5 (0.040) [0.000-0.080] | 0.850 |
| Stomach cancer | 3 (0.128) [0.000-0.309] | 4 (0.044) [0.000-0.101] | 0.246 |
| Colorectal cancer | 46 (0.693) [0.473-0.913] | 38 (0.563) [0.223-0.903] | 0.528 |
| Lung cancer | 19 (0.343) [0.149-0.537] | 22 (0.283) [0.140-0.426] | 0.617 |
| Pancreatic cancer | 3 (0.057) [0.000-0.154] | 1 (0.024) [0.000-0.072] | 0.473 |
| Thyroid cancer | 21 (0.515) [0.280-0.751] | 21 (0.238) [0.103-0.374] | 0.044 |
| Prostate cancer | 104 (2.121) [1.527-2.715] | 113 (1.308) [1.002-1.614] | 0.018 |
| Bladder cancer | 16 (0.297) [0.109-0.486] | 17 (0.172) [0.075-0.268] | 0.138 |
| Kidney cancer | 21 (0.335) [0.162-0.508] | 23 (0.249) [0.129-0.369] | 0.470 |
| Skin cancer | 145 (3.663) [2.965-4.361] | 187 (3.377) [2.816-3.938] | 0.520 |
| Hodgin Lymphoma | 19 (0.339) [0.140-0.539] | 15 (0.174) [0.061-0.286] | 0.127 |
| Leukaemia | 9 (0.148) [0.036-0.261] | 13 (0.184) [0.074-0.294] | 0.657 |
| Breast cancer | 116 (2.579) [2.076-3.082] | 109 (2.089) [1.502-2.676] | 0.158 |
| Cervical cancer | 26 (0.471) [0.244-0.698] | 34 (0.694) [0.376-1.013] | 0.179 |
| Ovarian cancer | 15 (0.419) [0.109-0.728] | 13 (0.285) [0.028-0.543] | 0.503 |
| Liver cancer | 3 (0.050) [0.000-0.122] | 2 (0.017) [0.000-0.042] | 0.261 |

Data were presented as unweighted number and weighted percentage with 95% confidence interval;

Supplemental Table 4. Causal effect of genetically predicted cALT on extraheptic cancers

| Cancer Types | No. of SNPs | IVW-random effects | |  | Weighted median | |  | MR-Egger | |
| --- | --- | --- | --- | --- | --- | --- | --- | --- | --- |
|  |  | OR (95%CI) | P |  | OR (95%CI) | P |  | OR (95%CI) | P |
| Esophagus cancer | 48 | 1.013 (0.880-1.166) | 0.859 |  | 1.215 (0.999-1.479) | 0.052 |  | 1.244 (0.987-1.569) | 0.071 |
| Stomach cancer | 49 | 0.994 (0.924-1.069) | 0.866 |  | 0.998 (0.896-1.112) | 0.971 |  | 1.067 (0.943-1.207) | 0.311 |
| Colorectal cancer | 48 | 1.001 (0.938-1.069) | 0.976 |  | 1.025 (0.941-1.116) | 0.571 |  | 0.968 (0.866-1.082) | 0.566 |
| Lung cancer | 49 | 1.020 (0.947-1.098) | 0.609 |  | 0.977 (0.872-1.095) | 0.691 |  | 0.979 (0.862-1.111) | 0.741 |
| Pancreatic cancer | 40 | 0.975 (0.829-1.148) | 0.763 |  | 0.943 (0.733-1.214) | 0.649 |  | 0.915 (0.695-1.204) | 0.529 |
| Thyroid cancer | 49 | 1.098 (0.946-1.274) | 0.218 |  | 1.092 (0.841-1.419) | 0.507 |  | 1.219 (0.924-1.609) | 0.168 |
| Prostate cancer | 48 | 0.992 (0.945-1.042) | 0.757 |  | 1.001 (0.944-1.061) | 0.977 |  | 0.948 (0.868-1.035) | 0.240 |
| Bladder cancer | 47 | 1.000 (0.999-1.000) | 0.252 |  | 1.000 (0.999-1.001) | 0.676 |  | 1.000 (0.999-1.001) | 0.400 |
| Kidney cancer | 39 | 1.118 (0.894-1.398) | 0.330 |  | 1.006 (0.771-1.313) | 0.965 |  | 1.006 (0.693-1.461) | 0.976 |
| Skin cancer | 47 | 0.972 (0.926-1.021) | 0.252 |  | 0.987 (0.925-1.053) | 0.694 |  | 1.038 (0.957-1.125) | 0.373 |
| Malignant Lymphoma | 48 | 0.964 (0.867-1.071) | 0.493 |  | 0.949 (0.813-1.108) | 0.506 |  | 1.027 (0.855-1.232) | 0.779 |
| Non-Hodgin Lymphoma | 38 | 1.008 (0.862-1.179) | 0.920 |  | 0.999 (0.815-1.224) | 0.989 |  | 1.133 (0.873-1.469) | 0.354 |
| Leukaemia | 47 | 1.001 (1.000-1.001) | 0.085 |  | 1.000 (0.999-1.001) | 0.520 |  | 1.000 (0.999-1.001) | 0.648 |
| Breast cancer | 45 | 0.998 (0.961-1.037) | 0.933 |  | 0.989 (0.945-1.035) | 0.630 |  | 0.951 (0.892-1.014) | 0.129 |
| Cervical cancer | 48 | 1.001 (1.000-1.003) | 0.078 |  | 1.000 (0.998-1.002) | 0.928 |  | 1.000 (0.998-1.003) | 0.748 |
| Endometrial cancer | 51 | 1.028 (0.940-1.126) | 0.544 |  | 1.023 (0.916-1.142) | 0.688 |  | 0.980 (0.838-1.146) | 0.797 |
| Ovarian cancer | 45 | 0.965 (0.912-1.020) | 0.209 |  | 0.945 (0.871-1.026) | 0.179 |  | 0.993 (0.900-1.095) | 0.885 |
| Liver & bile duct cancer | 48 | 1.001 (1.000-1.001) | 0.011 |  | 1.001 (1.000-1.001) | 0.003 |  | 1.001 (1.000-1.001) | 0.252 |

Abbreviations: cALT, chronically elevated serum alanine aminotransferase; SNPs, single nucleotide polymorphisms; IVW, inverse variance weighted; MR, mendelian randomization; OR, odd ratio; CI, confidence interval.

Supplemental Table 5. Causal effect of genetically predicted imaging-based and biopsy-confirmed NAFLD on extraheptic cancers

| Cancer Types | No. of SNPs | IVW-random effects | |  | Weighted median | |  | MR-Egger | |
| --- | --- | --- | --- | --- | --- | --- | --- | --- | --- |
|  |  | OR (95%CI) | P |  | OR (95%CI) | P |  | OR (95%CI) | P |
| Esophagus cancer | 12 | 1.027 (0.925-1.141) | 0.618 |  | 1.086 (0.998-1.181) | 0.055 |  | 1.158 (1.030-1.301) | 0.034 |
| Stomach cancer | 12 | 0.997 (0.964-1.032) | 0.866 |  | 0.996 (0.954-1.040) | 0.857 |  | 1.022 (0.967-1.080) | 0.463 |
| Colorectal cancer | 12 | 1.000 (0.963-1.039) | 0.991 |  | 1.015 (0.980-1.051) | 0.415 |  | 1.022 (0.969-1.079) | 0.443 |
| Lung cancer | 12 | 1.005 (0.973-1.037) | 0.778 |  | 0.989 (0.947-1.033) | 0.617 |  | 0.975 (0.919-1.033) | 0.408 |
| Pancreatic cancer | 11 | 0.963 (0.897-1.033) | 0.294 |  | 0.968 (0.871-1.075) | 0.541 |  | 0.935 (0.819-1.069) | 0.351 |
| Thyroid cancer | 12 | 1.026 (0.927-1.135) | 0.625 |  | 1.026 (0.918-1.146) | 0.651 |  | 0.983 (0.848-1.139) | 0.822 |
| Prostate cancer | 12 | 0.993 (0.961-1.026) | 0.674 |  | 0.972 (0.946-0.998) | 0.036 |  | 0.963 (0.923-1.004) | 0.107 |
| Bladder cancer | 11 | 1.000 (1.000-1.000) | 0.450 |  | 1.000 (0.999-1.000) | 0.870 |  | 1.000 (0.999-1.001) | 0.888 |
| Kidney cancer | 11 | 1.031 (0.940-1.130) | 0.522 |  | 1.007 (0.891-1.139) | 0.907 |  | 1.000 (0.856-1.167) | 0.998 |
| Skin cancer | 12 | 0.993 (0.973-1.015) | 0.533 |  | 0.997 (0.967-1.027) | 0.834 |  | 0.998 (0.962-1.036) | 0.922 |
| Malignant Lymphoma | 12 | 0.949 (0.903-0.998) | 0.040 |  | 0.961 (0.893-1.033) | 0.277 |  | 0.941 (0.861-1.028) | 0.206 |
| Non-Hodgin Lymphoma | 11 | 0.957 (0.872-1.051) | 0.362 |  | 0.975 (0.885-1.073) | 0.600 |  | 0.984 (0.855-1.132) | 0.825 |
| Leukaemia | 11 | 1.000 (0.999-1.000) | 0.842 |  | 1.000 (1.000-1.001) | 0.907 |  | 1.000 (1.000-1.001) | 0.747 |
| Breast cancer | 11 | 1.000 (0.972-1.028) | 0.980 |  | 1.000 (0.979-1.021) | 0.993 |  | 0.996 (0.956-1.038) | 0.856 |
| Cervical cancer | 12 | 1.000 (0.999-1.001) | 0.800 |  | 1.000 (0.999-1.001) | 0.691 |  | 0.999 (0.998-1.001) | 0.405 |
| Endometrial cancer | 12 | 1.009 (0.943-1.080) | 0.796 |  | 1.014 (0.967-1.063) | 0.575 |  | 0.982 (0.889-1.085) | 0.726 |
| Ovarian cancer | 11 | 1.000 (0.974-1.026) | 0.983 |  | 0.984 (0.942-1.028) | 0.463 |  | 0.981 (0.931-1.034) | 0.495 |
| Liver & bile duct cancer | 9 | 1.001 (1.000-1.001) | <0.001 |  | 1.000 (1.000-1.001) | 0.002 |  | 1.000 (1.000-1.001) | 0.061 |

Abbreviations: NAFLD, nonalcoholic fatty liver disease; SNPs, single nucleotide polymorphisms; IVW, inverse variance weighted; MR, mendelian randomization; OR, odd ratio; CI, confidence interval.

Supplemental Table 6. Sensitivity analyses of MR analysis of genetically predicted cALT on extrahepatic cancers

| Cancer Types | IVW-Cochran's Q test/Heterogeneity | |  | MR-Egger intercept/Pleiotropy | | |  | MR-PRESSO | | | | | |
| --- | --- | --- | --- | --- | --- | --- | --- | --- | --- | --- | --- | --- | --- |
|  | Q | P value |  | Intercept | SE | P value |  | Global test,  P value | Outliers | Beta* | SE* | P value* | Distortion test,  P value* |
| Esophagus cancer | 54.254 | 0.218 |  | -0.021 | 0.010 | 0.037 |  | 0.192 | NA | NA | NA | NA | NA |
| Stomach cancer | 48.105 | 0.469 |  | -0.007 | 0.005 | 0.171 |  | 0.509 | NA | NA | NA | NA | NA |
| Colorectal cancer | 67.699 | 0.026 |  | 0.003 | 0.005 | 0.464 |  | 0.027 | NA | NA | NA | NA | NA |
| Lung cancer | 52.441 | 0.306 |  | 0.004 | 0.005 | 0.440 |  | 0.324 | NA | NA | NA | NA | NA |
| Pancreatic cancer | 43.452 | 0.327 |  | 0.007 | 0.012 | 0.572 |  | 0.343 | NA | NA | NA | NA | NA |
| Thyroid cancer | 39.734 | 0.796 |  | -0.011 | 0.012 | 0.365 |  | 0.819 | NA | NA | NA | NA | NA |
| Prostate cancer | 90.703 | <0.001 |  | 0.004 | 0.004 | 0.227 |  | <0.001 | 2 | -0.006 | 0.021 | 0.790 | 0.882 |
| Bladder cancer | 42.849 | 0.605 |  | <0.001 | <0.001 | 0.800 |  | 0.625 | NA | NA | NA | NA | NA |
| Kidney cancer | 62.879 | 0.007 |  | 0.012 | 0.017 | 0.492 |  | 0.014 | 1 | 0.135 | 0.105 | 0.210 | 0.808 |
| Skin cancer | 68.056 | 0.019 |  | -0.007 | 0.003 | 0.056 |  | 0.022 | NA | NA | NA | NA | NA |
| Malignant Lymphoma | 56.391 | 0.164 |  | -0.007 | 0.008 | 0.409 |  | 0.165 | NA | NA | NA | NA | NA |
| Non-Hodgin Lymphoma | 54.794 | 0.030 |  | -0.013 | 0.012 | 0.278 |  | 0.032 | 1 | -0.068 | 0.079 | 0.396 | 0.369 |
| Leukaemia | 53.241 | 0.216 |  | <0.001 | <0.001 | 0.514 |  | 0.234 | NA | NA | NA | NA | NA |
| Breast cancer | 88.218 | <0.001 |  | 0.005 | 0.003 | 0.072 |  | <0.001 | 1 | -0.006 | 0.019 | 0.735 | 0.880 |
| Cervical cancer | 64.246 | 0.048 |  | 0.000 | 0.000 | 0.460 |  | 0.042 | 1 | 0.001 | 0.001 | 0.146 | 0.556 |
| Endometrial cancer | 102.498 | <0.001 |  | 0.005 | 0.006 | 0.458 |  | <0.001 | 1 | 0.017 | 0.043 | 0.696 | 0.491 |
| Ovarian cancer | 43.660 | 0.486 |  | -0.003 | 0.004 | 0.480 |  | 0.522 | NA | NA | NA | NA | NA |
| Liver & bile duct cancer | 74.579 | 0.006 |  | <0.001 | <0.001 | 0.871 |  | 0.007 | NA | NA | NA | NA | NA |

Abbreviations: cALT, chronically elevated serum alanine aminotransferase; IVW, inverse variance weighted; MR, mendelian randomization; MR-PRESSO, MR pleiotropy residual sum and outlier.

Supplemental Table 7. Sensitivity analyses of MR analysis of genetically predicted imaging-based and biopsy-confirmed NAFLD on extrahepatic cancers

| Cancer Types | IVW-Cochran's Q test/Heterogeneity | |  | MR-Egger intercept/Pleiotropy | | |  | MR-PRESSO | | | | | |
| --- | --- | --- | --- | --- | --- | --- | --- | --- | --- | --- | --- | --- | --- |
|  | Q | P value |  | Intercept | SE | P value |  | Global test,  P value | Outliers | Beta* | SE* | P value* | Distortion test,  P value* |
| Esophagus cancer | 21.339 | 0.030 |  | -0.049 | 0.018 | 0.018 |  | 0.168 | NA | NA | NA | NA | NA |
| Stomach cancer | 8.219 | 0.694 |  | -0.011 | 0.009 | 0.249 |  | 0.816 | NA | NA | NA | NA | NA |
| Colorectal cancer | 16.289 | 0.131 |  | -0.009 | 0.008 | 0.293 |  | 0.259 | NA | NA | NA | NA | NA |
| Lung cancer | 6.590 | 0.831 |  | 0.013 | 0.009 | 0.184 |  | 0.726 | NA | NA | NA | NA | NA |
| Pancreatic cancer | 5.851 | 0.828 |  | 0.012 | 0.020 | 0.568 |  | 0.853 | NA | NA | NA | NA | NA |
| Thyroid cancer | 11.082 | 0.437 |  | 0.017 | 0.022 | 0.451 |  | 0.592 | NA | NA | NA | NA | NA |
| Prostate cancer | 23.881 | 0.013 |  | 0.011 | 0.006 | 0.073 |  | 0.116 | NA | NA | NA | NA | NA |
| Bladder cancer | 9.514 | 0.484 |  | 0.000 | 0.000 | 0.380 |  | 0.548 | NA | NA | NA | NA | NA |
| Kidney cancer | 7.351 | 0.692 |  | 0.012 | 0.022 | 0.606 |  | 0.749 | NA | NA | NA | NA | NA |
| Skin cancer | 7.316 | 0.773 |  | -0.002 | 0.005 | 0.735 |  | 0.804 | NA | NA | NA | NA | NA |
| Malignant Lymphoma | 7.167 | 0.785 |  | 0.004 | 0.012 | 0.784 |  | 0.729 | NA | NA | NA | NA | NA |
| Non-Hodgin Lymphoma | 13.285 | 0.208 |  | -0.011 | 0.020 | 0.609 |  | 0.356 | NA | NA | NA | NA | NA |
| Leukaemia | 8.040 | 0.625 |  | 0.000 | 0.000 | 0.538 |  | 0.720 | NA | NA | NA | NA | NA |
| Breast cancer | 25.272 | 0.005 |  | 0.001 | 0.006 | 0.818 |  | 0.059 | NA | NA | NA | NA | NA |
| Cervical cancer | 14.062 | 0.230 |  | 0.000 | 0.000 | 0.175 |  | 0.328 | NA | NA | NA | NA | NA |
| Endometrial cancer | 28.907 | 0.002 |  | 0.010 | 0.014 | 0.473 |  | 0.026 | 1 | 0.004 | 0.031 | 0.889 | 0.387 |
| Ovarian cancer | 4.972 | 0.893 |  | 0.007 | 0.007 | 0.352 |  | 0.703 | NA | NA | NA | NA | NA |
| Liver & bile duct cancer | 9.065 | 0.337 |  | 0.000 | <0.001 | 0.237 |  | 0.426 | NA | NA | NA | NA | NA |

Abbreviations: NAFLD, nonalcoholic fatty liver disease; IVW, inverse variance weighted; MR, mendelian randomization; MR-PRESSO, MR pleiotropy residual sum and outlier.


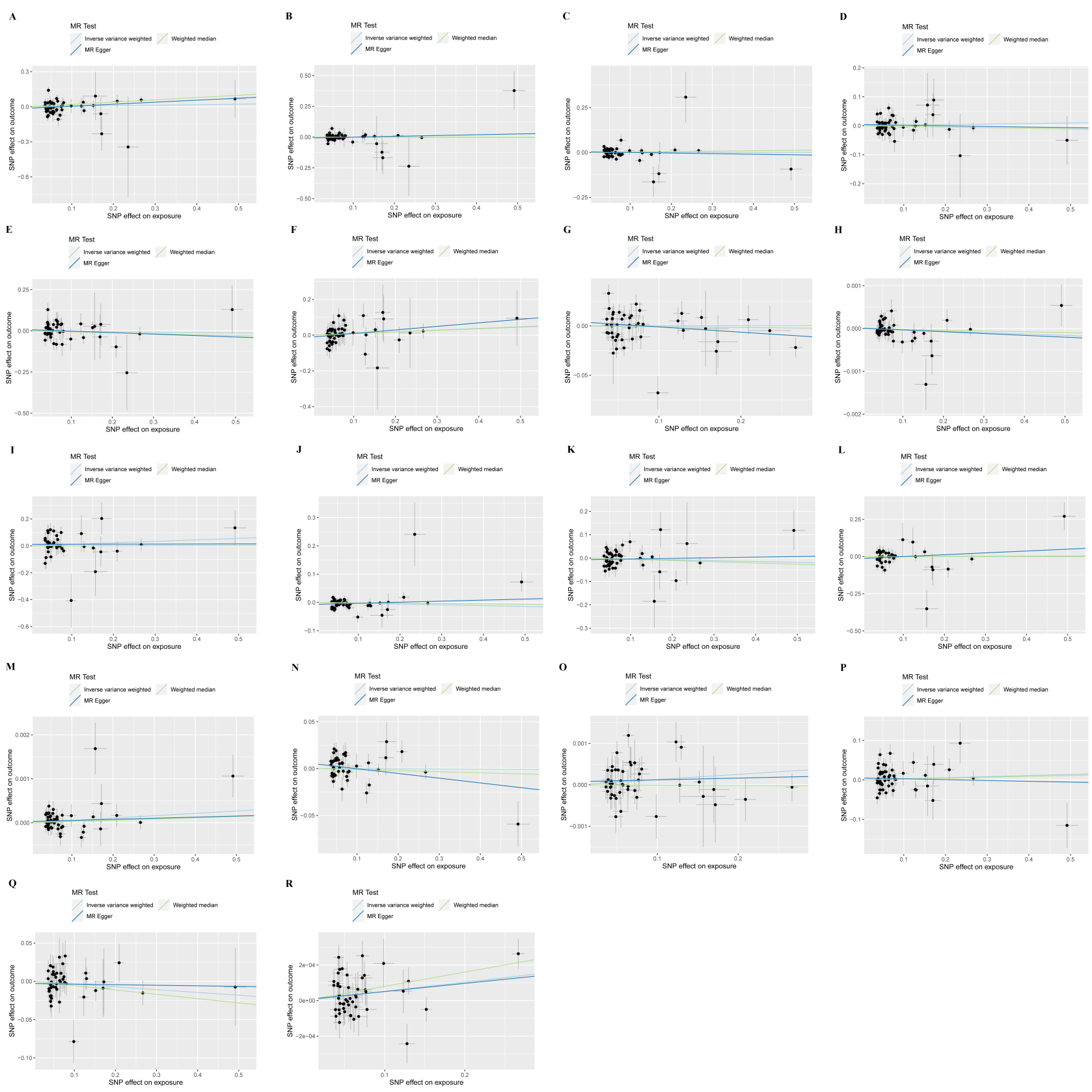


Supplemental Figure 1. Scatter plots of the causal effect of genetically predicted cALT on extrahepatic cancers. (A) Esophagus cancer; (B) Stomach cancer; (C) Colorectal cancer; (D) Lung cancer; (E) Pancreatic cancer; (F) Thyroid cancer; (G) Prostate cancer; (H) Bladder cancer; (I) Kidney cancer; (J) Skin cancer; (K) Malignant lymphoma; (L) NonHodgkin lymphoma; (M) Leukaemia; (N) Breast cancer; (O) Cervical cancer; (P) Endometrial cancer; (Q) Ovarian cancer; (R) Liver & bile duct cancer. Abbreviations: cALT, chronically elevated serum alanine aminotransferase levels.


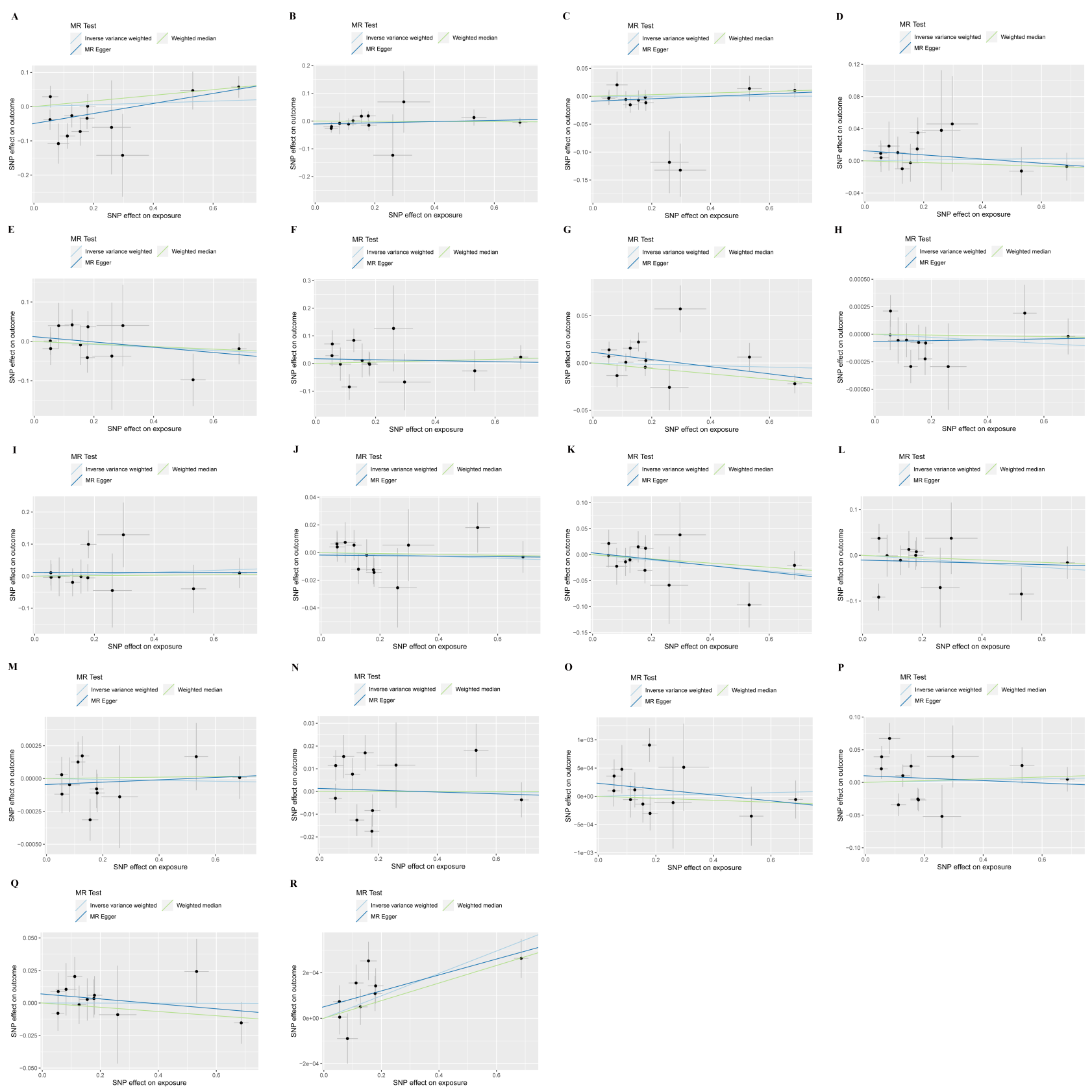


Supplemental Figure 2. Scatter plots of the causal effect of genetically predicted imaging-based and biopsy-confirmed NAFLD on extrahepatic cancers. (A) Esophagus cancer; (B) Stomach cancer; (C) Colorectal cancer; (D) Lung cancer; (E) Pancreatic cancer; (F) Thyroid cancer; (G) Prostate cancer; (H) Bladder cancer; (I) Kidney cancer; (J) Skin cancer; (K) Malignant lymphoma; (L) NonHodgkin lymphoma; (M) Leukaemia; (N) Breast cancer; (O) Cervical cancer; (P) Endometrial cancer; (Q) Ovarian cancer; (R) Liver & bile duct cancer. Abbreviations: NAFLD, nonalcoholic fatty liver disease.



Supplemental Figure 3: Funnel plots of the causal effect of genetically predicted cALT on extrahepatic cancers. (A) Esophagus cancer; (B) Stomach cancer; (C) Colorectal cancer; (D) Lung cancer; (E) Pancreatic cancer; (F) Thyroid cancer; (G) Prostate cancer; (H) Bladder cancer; (I) Kidney cancer; (J) Skin cancer; (K) Malignant lymphoma; (L) NonHodgkin lymphoma; (M) Leukaemia; (N) Breast cancer; (O) Cervical cancer; (P) Endometrial cancer; (Q) Ovarian cancer; (R) Liver & bile duct cancer. Abbreviations: cALT, chronically elevated serum alanine aminotransferase levels.


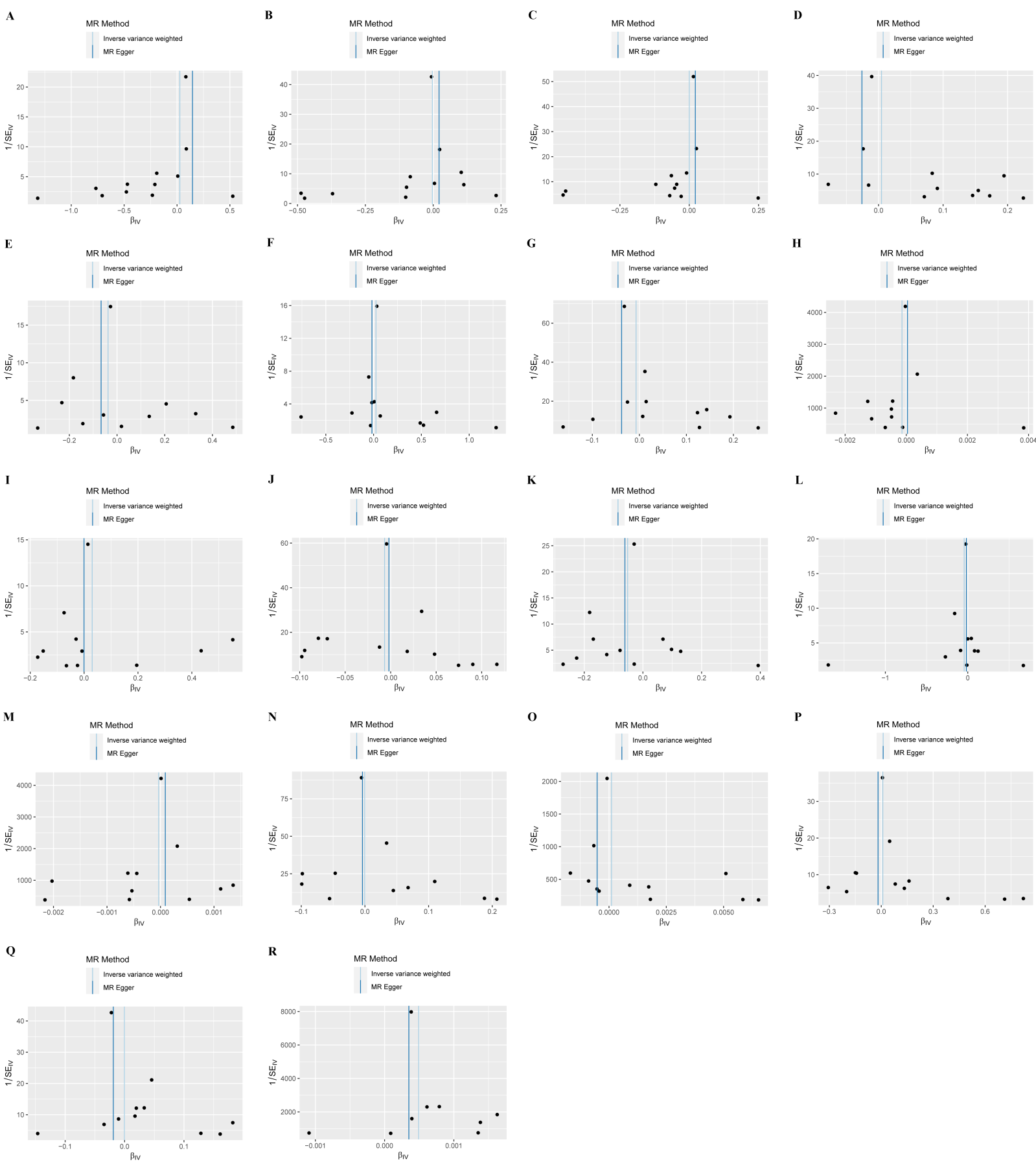
Supplemental Figure 4: Funnel plots of the causal effect of genetically predicted imaging-based and biopsy-confirmed NAFLD on extrahepatic cancers. (A) Esophagus cancer; (B) Stomach cancer; (C) Colorectal cancer; (D) Lung cancer; (E) Pancreatic cancer; (F) Thyroid cancer; (G) Prostate cancer; (H) Bladder cancer; (I) Kidney cancer; (J) Skin cancer; (K) Malignant lymphoma; (L) NonHodgkin lymphoma; (M) Leukaemia; (N) Breast cancer; (O) Cervical cancer; (P) Endometrial cancer; (Q) Ovarian cancer; (R) Liver & bile duct cancer. Abbreviations: NAFLD, nonalcoholic fatty liver disease.


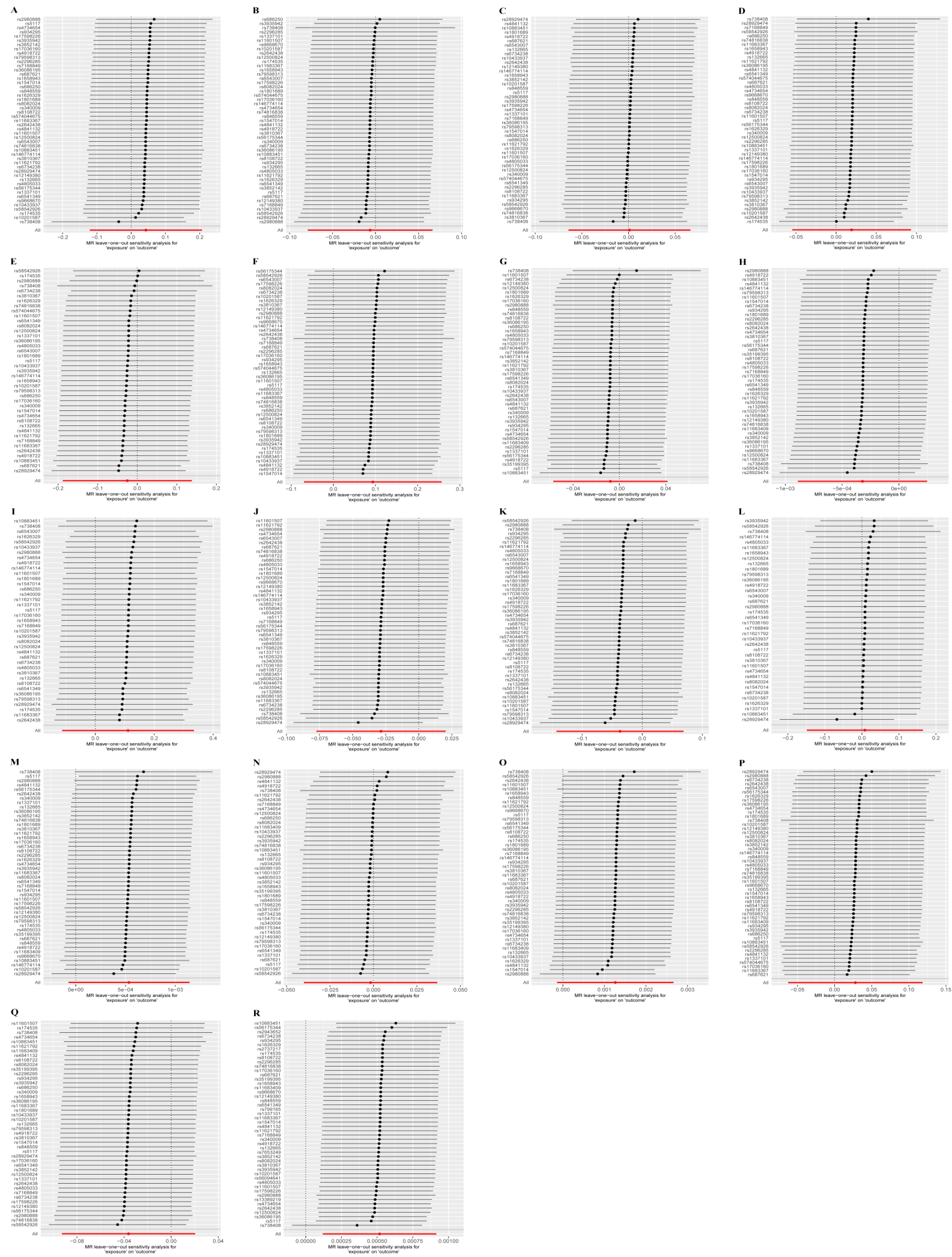
Supplemental Figure 5: Leave-one-out analyses for genetically predicted cALT on extrahepatic cancers. (A) Esophagus cancer; (B) Stomach cancer; (C) Colorectal cancer; (D) Lung cancer; (E) Pancreatic cancer; (F) Thyroid cancer; (G) Prostate cancer; (H) Bladder cancer; (I) Kidney cancer; (J) Skin cancer; (K) Malignant lymphoma; (L) NonHodgkin lymphoma; (M) Leukaemia; (N) Breast cancer; (O) Cervical cancer; (P) Endometrial cancer; (Q) Ovarian cancer; (R) Liver & bile duct cancer. Abbreviations: NAFLD, nonalcoholic fatty liver disease..


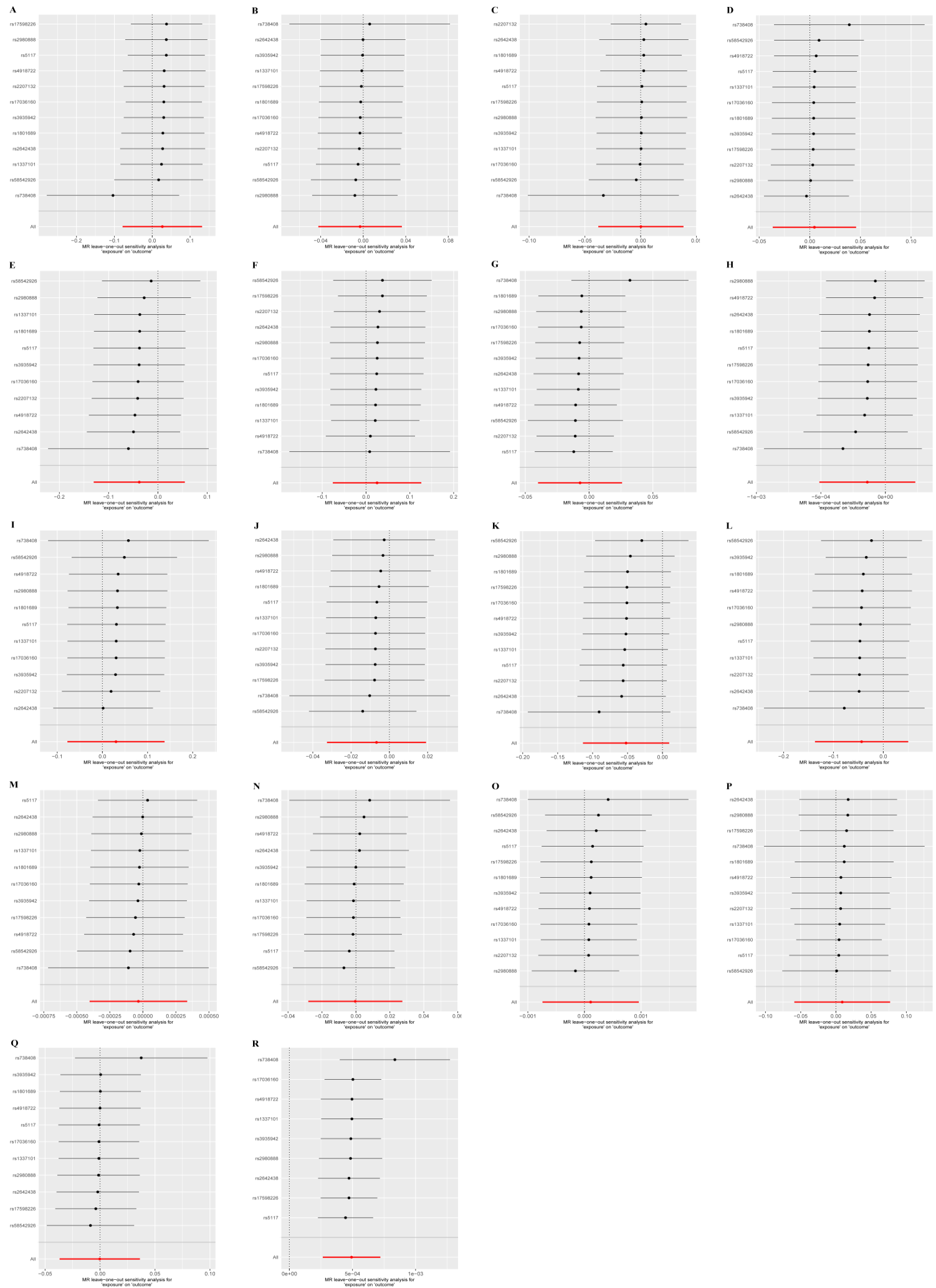
Supplemental Figure 6: Leave-one-out analyses for genetically predicted imaging-based and biopsy-confirmed NAFLD on extrahepatic cancers. (A) Esophagus cancer; (B) Stomach cancer; (C) Colorectal cancer; (D) Lung cancer; (E) Pancreatic cancer; (F) Thyroid cancer; (G) Prostate cancer; (H) Bladder cancer; (I) Kidney cancer; (J) Skin cancer; (K) Malignant lymphoma; (L) NonHodgkin lymphoma; (M) Leukaemia; (N) Breast cancer; (O) Cervical cancer; (P) Endometrial cancer; (Q) Ovarian cancer; (R) Liver & bile duct cancer. Abbreviations: NAFLD, nonalcoholic fatty liver disease.
